# Expression of ERβ, ERα and Her-2 and distribution of molecular subtypes in Uygur and Han patients with breast cancer

**DOI:** 10.3892/etm.2014.1596

**Published:** 2014-03-04

**Authors:** LIYING GUO, XUEQIN HOU, YILAMU DILIMINA, BOWEI WANG

**Affiliations:** 1 Department of Breast Cancer, Digestive and Vascular Center, The First Affiliated Hospital of Xinjiang Medical University, Urumchi, Xingjiang 830054, P.R. China; 2 Department of Pathology, The First Affiliated Hospital of Xinjiang Medical University, Urumchi, Xingjiang 830054, P.R. China

**Keywords:** breast cancer, ethnicity, estrogen receptor α, estrogen receptor β, human epidermal growth factor receptor-2, molecular subtypes

## Abstract

The aim of the present study was to investigate the expression levels of estrogen receptor (ER) α and β and human epidermal growth factor receptor-2 (Her-2), as well as the distribution of breast cancer molecular subtypes in Uygur and Han breast cancer patients. Cancer tissues were collected and analyzed from 709 breast cancer patients enrolled between January 2000 and December 2010. The expression levels of ERα, ERβ and Her-2 were determined by immunohistochemistry. The differences in expression and molecular subtype distribution between Uygur and Han patients with breast cancer were analyzed using a Pearson’s χ^2^ test. Statistically significant differences were observed in the expression levels of ERβ, ERα and Her-2 between the Uygur and Han patients with breast cancer (P<0.05). The expression levels of ERβ (++), ERβ (+++) and Her-2 (+++) in Uygur patients were significantly higher, while the expression levels of ERα (+++) in Uygur patients were significantly lower when compared with Han patients. The percentage of luminal A type breast cancer in Uygur patients was significantly lower when compared with the Han patients, whereas the percentages of basal-like and Her-2 overexpression types were significantly higher than those in Han patients (P<0.05). Therefore, expression levels of ERβ, ERα and Her-2, and molecular subtypes of breast cancer were significantly different between the Uygur and Han populations.

## Introduction

Breast cancer is a tumor with heterogeneity, which differs among ethnic groups and individuals (1). Ethnicity has been reported to be an independent factor affecting the prognosis of breast cancer (2). Estrogen receptor (ER) β, the newly identified ER subtype, has potentially important clinical value for the research of biological characteristics and prognosis evaluation of breast cancer. ERα and human epidermal growth factor receptor-2 (Her-2) are commonly used immunohistochemistry indicators in clinical practice. These receptors are important for the guidance of postoperative chemotherapy and endocrine therapy of breast cancer, as well as for the evaluation of breast cancer prognosis. Breast cancer may be divided into various molecular subtypes based on the expression levels of ERβ, progesterone receptor and Her-2. Different molecular subtypes among various ethnicities have their own characteristics (3). In the present study, the expression levels of ERβ, ERα and Her-2, as well as the distributions of various molecular subtypes, were compared between Uygur and Han patients with breast cancer in Xinjiang, China.

## Materials and methods

### Patient data

A total of 730 specimens from patients with pathologically confirmed invasive breast cancers were used in the study. Twenty-one cases were censored, since certain patients succumbed to other causes or were lost to follow-up. The patients were diagnosed and underwent surgery in the First Affiliated Hospital of Xinjiang Medical University (Urumchi, China) between January 2000 and December 2010. The tumors were pathologically confirmed as clinical stage I–II invasive non-specific breast cancer. The clinical data were complete (Table I). Among the cases, there were 446 Han patients and 263 Uygur patients. The patients were followed-up for 2–10 years and 21 cases were censored. The patients that succumbed to other causes or were lost to follow-up at the time of last contact or prior to the study cut-off were censored. According to breast cancer molecular subtypes, 519 cases were selected, including 188 cases of luminal A (36.2%; ERα^+^, PR^+^, Her-2^−^), 128 cases of luminal B (24.7%; ERα^+^, PR^+^, Her-2^+^), 97 cases of Her-2 overexpression (18.7%; ERα^−^, PR^−^, Her-2^+^) and 106 cases of basal-like types (20.4%; ERα ^−^, PR^−^, Her-2^−^).

Prior written and informed consent was obtained from every patient and the study was approved by the Ethics Review Board of Xinjiang Medical University.

### Immunohistochemistry

Breast cancer tissue samples were fixed with 10% formaldehyde for 24 h, embedded in paraffin and sliced into 3-μm thick sections. Following dewaxing with xylene, the sections were treated with antigen retrieval reagents. After blocking, the sections were incubated with primary antibodies at 37°C in the dark for 1 h. The samples were then washed with phosphate-buffered saline (PBS) and secondary antibodies were added and incubated in the dark for 30 min. The sections were developed with 3,3′-diaminobenzidine chromogenic reagent. Finally, the sections were counterstained with hematoxylin and eosin. A positive sample was set up as a positive control and PBS, instead of primary antibody, was used as a negative control. ERβ antibodies and the working solution were purchased from Fuzhou Maixin Biotechnology Development Co., Ltd. (Fuzhou, China). ERα and Her-2 antibodies were obtained from Gene Tech (Shanghai) Co., Ltd. (Shanghai, China). Primary ERβ rabbit anti-human polyclonal antibody and HRP-polymer rabbit anti-mouse antibody were purchased from Yueyan Biotech Company (Shanghai, China). Primary ERα rabbit anti-human monoclonal antibody, primary PR rabbit anti-human monoclonal antibody, primary Her-2 rabbit anti-human monoclonal antibody and HRP-polymer rabbit anti-human antibody were purchased from ZSGB Biotech Company (Beijing, China).

### Determination of expression levels

For the determination of ERβ expression, cells with brown or yellow staining in the nucleus were considered as ERβ-positive cells. ERβ-positive cells were then counted. The ERβ-positive rate was the ratio of the number of ERβ-positive cells to the total number of cells. An ERβ-positive rate of <1% was defined as ERβ (−), a positive rate between 1 and 10% was defined as ERβ (+), an ERβ-positive rate between >10 and 50% was defined as ERβ (++) and an ERβ-positive rate of >50% was ERβ (+++).

For the determination of ERα expression, cells with brown or yellow particles in the nucleus were considered as ERα-positive cells. Expression levels of ERα were divided into four levels: ERα (−), positive rate <30%; ERα (+), positive rate between 30 and 40%; ERα (++), positive rate between >40 and 60%; ERα (+++), positive rate >60% (4).

For the determination of Her-2 expression, the Her-2 Detection Guide published by the Chinese Journal of Pathology in 2009 was used (5). Her-2 expression was defined as follows: Her-2 (−), no staining; Her-2 (+), weak or incomplete cell membrane staining; Her-2 (++), >10% of invasive cancer cells showing weak to moderate intensity with complete but non-uniform membrane staining or <30% of invasive cancer cells showing; Her-2 (++), >30% of invasive cancer cells showing strong, complete and uniform membrane staining.

### Statistical analysis

Data were analyzed using SPSS 17.0 (SPSS, Inc., Chicago, IL, USA) and differences were compared using a χ^2^ test. P<0.05 was considered to indicate a statistically significant difference.

## Results

### Expression levels of ERβ, ERα and Her-2 in breast cancer

**Figure 1:**
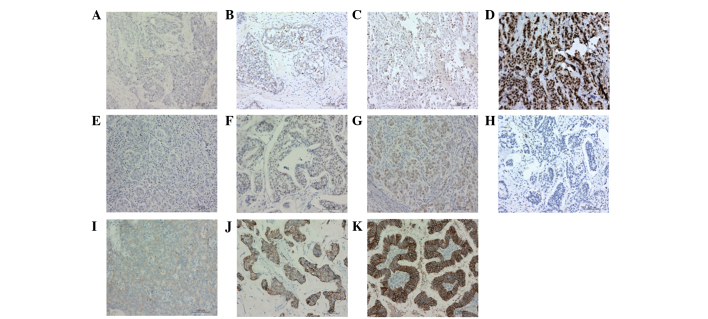
Expression of ERβ, ERα and Her-2 in breast cancer tissue. Expression of ERβ, ERα and Her-2 was determined by immunohistochemistry. Representative images were shown. Cells with brown staining were positive cells and cells with blue staining were negative cells. Magnification, ×400. (A–D) Stained with ERβ antibody. (E–G) Stained with ERα antibody. (H–K) Stained with HER2 antibody.

To determine the expression levels of ERβ, ERα and Her-2 in breast cancer tissue, immunohistochemical staining was performed. Representative results are shown in Fig. 1. Cells with blue staining were classified as negative expression cells and cells with brown staining were classified as positive expression cells. ERβ expression levels were divided into ERβ (−), (+), (++) and (+++) (Fig. 1A–D, respectively). ERα expression levels were divided into ERα (−), (+/++ ) and (+++) (Fig. 1E–G, respectively). Her-2 expression levels were divided into Her-2 (−), (+), (++) and (+++) (Fig. 1H–K, respectively). Molecular subtypes of breast cancer were defined based on the expression levels of ERα and Her-2.

### Positive ERβ expression is higher in Uygur breast cancer patients

To compare the difference in ERβ expression levels between Uygur and Han patients, a χ^2^ test was performed. The results are shown in Table II. The percentage of ERβ (−) in Uygur patients (47.5%) was lower than that in Han patients (57.2%). However, percentages of ERβ (+), (++) and (+++) in Uygur patients were 29.3, 11.8 and 11.4%, respectively, which were higher than those in Han patients (27.8, 8.5 and 6.5%, respectively). Statistically, the ERβ-positive expression rate in Uygur patients was significantly higher when compared with Han patients (P<0.05). This result indicates that Uygur patients exhibited higher levels of ERβ expression.

### Positive ERα expression is lower in Uygur breast cancer patients

Differences in ERα expression levels between Uygur and Han patients were also compared with a χ^2^ test. As shown in Table III, in Uygur patients, the percentage of ERα (−) expression (48.7%) was higher than that in Han patients (39.0%), whereas percentages of ERα (+/++) and (+++) expression (22.1 and 29.3%, respectively) were lower when compared with Han patients (24.9 and 36.15%, respectively). Statistically significant differences were observed in ERα expression levels between Uygur and Han patients (P<0.05). This result demonstrated that Uygur patients had lower levels of ERα expression.

### Positive Her-2 expression is higher in Uygur breast cancer patients

Her-2 expression, an additional immunohistochemical indicator of breast cancer, was further analyzed. The difference in Her-2 expression levels between Uygur and Han individuals was similar to ERβ expression (Table IV). In Uygur patients, the percentage of Her-2 (−) was 49.8%, which was lower than in Han patients (58.3%). The percentages of Her-2 (+), (++) and (+++) in Uygur patients were 11.4, 9.9 and 28.9%, respectively, which were higher than those in Han patients (13.0, 6.5 and 22.2%, respectively). The differences between Uygur and Han patients were statistically significant (P<0.05), thus, the results indicated that Uygur patients had higher levels of Her-2 expression.

### Differences in molecular subtypes between Uygur and Han patients

As previously described, there are four molecular subtypes of breast cancer, including luminal A, luminal B, Her-2 overexpression and basal-like types. The distributions of these molecular subtypes in Uygur and Han patients were further investigated. As shown in Table V, when compared with Han patients, Uygur patients had a lower percentage of luminal A (33.3 vs. 37.8%) and luminal B types (20.2 vs. 27.1%), however, had higher levels of Her-2 overexpression (25.1 vs. 15.2%) and basal-like types (21.3 vs. 19.9%). These differences between Uygur and Han patients were statistically significant (P<0.05). Thus, the distributions of molecular subtypes in Uygur breast cancer patients were different from those in Han breast cancer patients.

## Discussion

In the present study, the differences among ERβ, ERα and Her-2 expression levels, as well as breast cancer molecular subtype distribution, in Uygur and Han patients were compared.

ERβ was first identified in mice and rats in 1996 (6). In 1997, Dotzlaw *et al* (7) observed the expression levels of ERβ in human tumor tissues. Since then, the clinical significance of ERβ expression has gained much attention. Järvinen *et al* (8) detected 55 cases of ERβ expression out of 92 cases (59.8%) of breast cancer; Mann *et al* (9) detected 78 cases (66%) out of 118 cases of breast cancer; Fuqua *et al* (10) detected 184 cases (76%) out of 242 cases; Han *et al* (11) detected 66 cases (42.6%) out of 155 cases. However, there are few studies investigating the difference in ERβ expression between Uygur and Han populations. In the present study, ERβ expression levels were compared in 263 Uygur and 446 Han breast cancer patients. The results demonstrated that the percentages of ERβ (−) in Uygur and Han patients were 47.5 and 57.2%, respectively, which was statistically significant (P<0.05). In addition, ERβ-positive expression levels in Uygur patients were significantly higher when compared with Han patients (P<0.05). It has been reported that in other tumors, including esophageal (12) and cervical cancers (13), incidence and gene expression differ among various ethnic groups. These differences may be associated with genetic background, environmental factors, lifestyle, diet and cultural level. The results of the present study indicated that there was also a difference in ERβ expression in breast cancer between Uygur and Han patients.

ERα is an important indicator of breast cancer prognosis and endocrine therapy. Elledge *et al* (14) studied ERα expression in females of various ethnicities, including African-American and Caucasian. The results demonstrated that the levels of ERα expression were higher in Caucasian and Asian females, while lower in African females. The levels of ERα expression in Hispanic females were intermediate (14,15). These observations indicate that ERα expression exhibits ethnic differences. In the present study, differences were also identified in ERα expression levels between Uygur and Han patients. The ERα (+++) rate was 29.3% in Uygur patients, which was significantly lower than that in Han patients (36.1%). Schwartz *et al* (16) observed that the ERα-positive expression rate of Chinese females with primary breast cancer was lower than that of European and American females, indicating that ERα expression may also be associated with various regions and ethnic factors. Therefore, these observations further indicate that the estrogen-sensitivity of breast cancer patients in Uygur individuals may be lower than that in Han individuals, which may be one of the reasons for the different therapeutic effects of breast cancer in Uygur and Han individuals.

Her-2, a proto-oncogene, is a common breast cancer gene marker that is involved in the regulation of cell growth, proliferation and differentiation. Overexpression of Her-2 often indicates a high degree of malignancy. Al-Abbadi *et al* (17) compared Her-2 expression levels in breast cancer between Caucasian-Americans and African-Americans. The authors observed that there was no significant difference between the two ethnicities. By contrast, Yang *et al* (18) observed that the Her-2 expression rate in breast infiltration ductal carcinoma tissues of Han patients was significantly higher than in Uygur patients, indicating that Her-2 expression exhibited ethnic differences. Thus, whether Her-2 is differentially expressed in different ethnicities remains controversial. In the present study, Her-2 expression was compared between Uygur and Han individuals and a significant difference was observed. The Her-2 (+++) expression rate (28.9%) in Uygur individuals was significantly higher when compared with Han individuals (22.2%; P<0.05). However, whether the difference was caused by inherent differences in inter-ethnic genomes or the results of different expression progression requires further investigation (18).

Based on the differences observed in gene expression levels in tumor tissues, Perou *et al* (19) divided breast cancer into five molecular subtypes in 2000, which included luminal A, luminal B, Her-2 overexpression and basal-like types. Different subtypes had different prognoses. Carey *et al* (20) examined the immunohistochemistry indicators of Caucasian and African-American females with breast cancer from 24 towns and cities in eastern and central North Carolina. The results demonstrated that there was an ethnic difference in basal-like type distribution in this population. Carey *et al* (20) compared the molecular subtypes of African-Americans and non-African-Americans. The authors observed that the expression levels of basal-like and luminal A types in the two ethnicities differed. In addition, the study revealed that Her-2 overexpression and basal-like types had a higher histological grade and greater cell proliferation capability as compared with other subtypes of breast cancer, indicating poor prognosis of the Her-2 overexpression and basal-like types. The authors also found that luminal A type cases had a better prognosis than other subtypes. However, the differences in breast cancer subtypes of Uygur and Han individuals in Xinjiang were not reported. In the present study, the distribution of various breast cancer molecular subtypes were compared in Uygur and Han patients. Luminal A type was identified to be the main molecular subtype in Uygur and Han individuals (33.3 and 37.8%, respectively), which was similar to the results of a previous study (16). The percentage of luminal A type in Uygur patients was lower than that in Han patients. However, the percentages of basal-like and Her-2 overexpression types in Uygur individuals were higher than those in Han individuals, and the difference was statistically different (P<0.05). Thus, the results of the present study indicate that breast cancer in Uygur individuals has a higher degree of malignancy than that in Han individuals.

In conclusion, in Xinjiang, the expression levels of ERβ and Her-2 and the percentages of basal-like and Her-2 overexpression types in Uygur individuals were higher than those in Han individuals. The expression levels of ERα and the percentages of luminal A and luminal B types in Uygur individuals were lower than those in Han individuals. These observations indicate that breast cancer in Uygur patients may have a higher degree of malignancy and poorer prognosis than that of Han patients. However, the underlying mechanisms of these differences require further study. The results of the present study provide experimental evidence for evaluating prognosis and developing individualized comprehensive treatment for Uygur and Han patients with breast cancer.

## Figures and Tables

**Table I:** Clinical data of Uygur and Han breast cancer patients.

Clinical features	Cases, n (%)
Ethnicity	
Uygur	263 (37.1)
Han	446 (62.9)
Age, years	
≤49	377 (53.2)
≥50	332 (46.8)
Tumor size, cm	
≤2	257 (36.2)
>2≤5	352 (49.6)
>5	100 (14.1)
Histological grade	
Grade I	125 (17.6)
Grade II	396 (55.9)
Grade III	188 (26.5)
Pathological stage	
Stage I	312 (44.0)
Stage II	397 (56.0)
Lymph node metastasis	
Negative	412 (58.1)
Positive	297 (41.9)
ERβ expression	
(−)	380 (53.6)
(+)	201 (28.3)
(++)	69 (9.7)
(+++)	59 (8.3)
ERα expression	
(−)	302 (42.6)
(+/++)	169 (23.8)
(+++)	238 (33.6)
Her-2 expression	
(−)	391 (55.1)
(+)	88 (12.4)
(++)	55 (7.8)
(+++)	175 (24.7)
Molecular subtype	
Luminal A	188 (26.5)
Luminal B	128 (18.1)
Her-2 overexpression	97 (13.7)
Basal-like	106 (15.0)

ER, estrogen receptor; Her-2, human epidermal growth factor receptor-2.

**Table II:** Comparison between ERβ expression levels in Uygur and Han breast cancer patients.

ERβ expression	Uygur, n (%)	Han, n (%)	χ^2^ value	P-value
(−)	125 (47.5)	255 (57.2)	9.596	0.022^a^
(+)	77 (29.3)	124 (27.8)		
(++)	31 (11.8)	38 (8.5)		
(+++)	30 (11.4)	29 (6.5)		

a P<0.05, Pearson’s χ^2^ test.

ER, estrogen receptor.

**Table III:** Comparison between ERα expression levels in Uygur and Han breast cancer patients.

ERα expression	Uygur, n (%)	Han, n (%)	χ^2^ value	P-value
(−)	128 (48.7)	174 (39.0)	6.472	0.039^a^
(+/++)	58 (22.1)	111 (24.9)		
(+++)	77 (29.3)	161 (36.1)		

a P<0.05, Pearson’s χ^2^ test.

ER, estrogen receptor.

**Table IV:** Comparison between Her-2 expression levels in Uygur and Han breast cancer patients.

Her-2 expression	Uygur, n (%)	Han, n (%)	χ^2^ value	P-value
(−)	131 (49.8)	260 (58.3)	7.951	0.047^a^
(+)	30 (11.4)	58 (13.0)		
(++)	26 (9.9)	29 (6.5)		
(+++)	76 (28.9)	99 (22.2)		

a P<0.05, Pearson’s χ^2^ test.

Her-2, human epidermal growth factor receptor 2.

**Table V:** Comparison between molecular subtypes in Uygur and Han breast cancer patients.

Molecular subtype	Uygur, n (%)	Han, n (%)	χ^2^ value	P-value
Luminal A	61 (33.3)	127 (37.8)	9.092	0.028^a^
Luminal B	37 (20.2)	91 (27.1)		
Her-2 overexpression	46 (25.1)	51 (15.2)		
Basal-like	39 (21.3)	67 (19.9)		

a P<0.05, Pearson’s χ^2^ test.

Her-2, human epidermal growth factor receptor 2.
